# Progressive neurodegeneration following spinal cord injury: Implications for clinical trials

**DOI:** 10.1212/WNL.0000000000005258

**Published:** 2018-04-03

**Authors:** Gabriel Ziegler, Patrick Grabher, Alan Thompson, Daniel Altmann, Markus Hupp, John Ashburner, Karl Friston, Nikolaus Weiskopf, Armin Curt, Patrick Freund

**Affiliations:** From the Institute of Cognitive Neurology and Dementia Research (G.Z.), Otto-von-Guericke-University Magdeburg; German Center for Neurodegenerative Diseases (G.Z.), Magdeburg, Germany; Spinal Cord Injury Center Balgrist (P.G., M.H., A.C., P.F.), University Hospital Zurich, University of Zurich, Switzerland; Department of Brain Repair & Rehabilitation (A.T., P.F.) and Wellcome Trust Centre for Neuroimaging (J.A., K.F., N.W., P.F.), UCL Institute of Neurology, UCL, London; Queen Square Multiple Sclerosis Centre (D.A.), Institute of Neurology, University College London; Medical Statistics Department (D.A.), London School of Hygiene & Tropical Medicine, London, UK; and Department of Neurophysics (N.W., P.F.), Max Planck Institute for Human Cognitive and Brain Sciences, Leipzig, Germany.

## Abstract

**Objective:**

To quantify atrophy, demyelination, and iron accumulation over 2 years following acute spinal cord injury and to identify MRI predictors of clinical outcomes and determine their suitability as surrogate markers of therapeutic intervention.

**Methods:**

We assessed 156 quantitative MRI datasets from 15 patients with spinal cord injury and 18 controls at baseline and 2, 6, 12, and 24 months after injury. Clinical recovery (including neuropathic pain) was assessed at each time point. Between-group differences in linear and nonlinear trajectories of volume, myelin, and iron change were estimated. Structural changes by 6 months were used to predict clinical outcomes at 2 years.

**Results:**

The majority of patients showed clinical improvement with recovery stabilizing at 2 years. Cord atrophy decelerated, while cortical white and gray matter atrophy progressed over 2 years. Myelin content in the spinal cord and cortex decreased progressively over time, while cerebellar loss decreases decelerated. As atrophy progressed in the thalamus, sustained iron accumulation was evident. Smaller cord and cranial corticospinal tract atrophy, and myelin changes within the sensorimotor cortices, by 6 months predicted recovery in lower extremity motor score at 2 years. Whereas greater cord atrophy and microstructural changes in the cerebellum, anterior cingulate cortex, and secondary sensory cortex by 6 months predicted worse sensory impairment and greater neuropathic pain intensity at 2 years.

**Conclusion:**

These results draw attention to trauma-induced neuroplastic processes and highlight the intimate relationships among neurodegenerative processes in the cord and brain. These measurable changes are sufficiently large, systematic, and predictive to render them viable outcome measures for clinical trials.

Recovery from spinal cord injury (SCI)—and its attendant neurodegenerative processes—follows a complex trajectory evolving over several years^1^ after trauma, where the ensuing neurodegeneration affects the spinal cord and brain.^2^ Given potential treatments that target repair of the injured spinal cord,^3^ there is an imperative to improve clinical trial design and efficiency, optimize patient stratification (in the context of disease heterogeneity), and identify trial outcome measures with predictive validity.^4^

Alterations in structure and function in motor, sensory, and limbic systems above the level of injury have been associated with impaired motor^5^ and sensory^6^ processes and neuropathic pain^7^ following SCI. Serial MRI has shown rapid, continuous volumetric and diffusivity changes in these systems^5–8^ above the injury following acute SCI. The available evidence suggests that reduction in myelin after 1 year accompanies atrophy and is associated with clinical impairment.^5^ By extending our longitudinal study to 2 years, we hoped to (1) establish that macroscopic volume changes continue with distinct trajectories; (2) characterize the associated demyelination^9^ and iron accumulation^10^; and (3) establish that neurodegenerative change within the first 6 months after injury predicts 2-year outcome.

We applied computational neuroimaging approaches to quantify volumetric changes in macroscopic tissue compartments^11^ and measures of myelination (via magnetization transfer saturation^12^ [MT]) and iron content (using the effective transverse relaxation rate^13^ [R2*]) over the course of 2 years. To characterize structural trajectories, we modeled the MRI measures and recovery in terms of linear rate of change (i.e., degeneration and recovery) and nonlinear changes in the rate (i.e., acceleration and deceleration). Structural MRI changes at 6 months were then used to predict 2-year outcome.

## Methods

### Participants and study design

Fifteen patients with traumatic SCI—admitted consecutively after surgical decompression into the rehabilitation program (1–2 weeks post injury) at the University Hospital Balgrist (Zurich, Switzerland) between September 2010 and July 2015—and 18 controls (table e-1, links.lww.com/WNL/A315) participated in a 2-year longitudinal study. Eligible patients with a traumatic SCI (<2 months post injury) and controls were older than 18 years, and had no history of head and brain lesions, no preexisting neurologic, mental, or medical disorders affecting outcome, and no contraindications to MRI. In total, 156 MRI datasets were analyzed from 33 (15 with SCI, 18 healthy controls) participants acquired at baseline and 2, 6, 12, and 24 months after SCI or study inclusion for controls, respectively (e-Methods, links.lww.com/WNL/A316). Follow-ups were performed successfully in 80.0%, 93.3%, 93.3%, and 86.7% (93.3% for clinical assessments) of patients, and 94.4%, 100.0%, 100.0%, and 100.0% of controls, respectively. In short, 94.6% of the participants completed follow-up. The last time point was delayed (up to 36 months) in a few patients but included in the analysis. All patients were assessed clinically using the International Standards for Neurological Classification of SCI protocol^14^ for motor, light-touch, and pinprick score and the Spinal Cord Independence Measure (SCIM). We assessed multiple aspects of pain (e.g., onset, duration [years], maximal and average pain intensity, quality of pain [e.g., nociceptive or neuropathic]) at each time point using a pain questionnaire (v4.2, emsci.org). The mean age difference between patients and controls was not found to be different (*p* = 0.0601, Mann–Whitney *U* test), but a sex imbalance was evident (*p* = 0.0342, Mann–Whitney *U* test). Both of these potentially confounding covariates were included in all statistical tests.

### Standard protocol approvals, registrations, and patient consents

All participants gave informed written consent and the study was approved by the local ethics committee of Zurich (EK-2010-0271).

### Structural image acquisition

We used a 3T Magnetom Verio (Siemens Healthcare, Munich, Germany) for the first 4 time points. Before acquiring the fifth time point of all subjects, the scanner was upgraded to a 3T Magnetom Skyra^fit^. The MRI protocol comprised a 3-dimensional (3D) whole-brain and cervical cord structural volume using an optimized, high-resolution, T1-weighted, 3D, MPRAGE (magnetization-prepared rapid-acquisition gradient echo) sequence and a multiparametric mapping protocol based on multiecho 3D FLASH (fast low angle shot) sequences (further acquisition details are provided in e-Methods, links.lww.com/WNL/A316). To assess the reproducibility of quantitative MRI measures across scanning sites, Weiskopf et al.^15^ (2013) established that regional intersite coefficient of variation of MT was smaller than 8%, while R2* was found to be less than 20%. The current single-site study used comparable techniques, suggesting that our quantitative MRI measures would be equally reliable, if not better.

### Longitudinal image processing and analysis

#### Neurodegeneration within the cervical cord over 2 years

**Figure 1:**
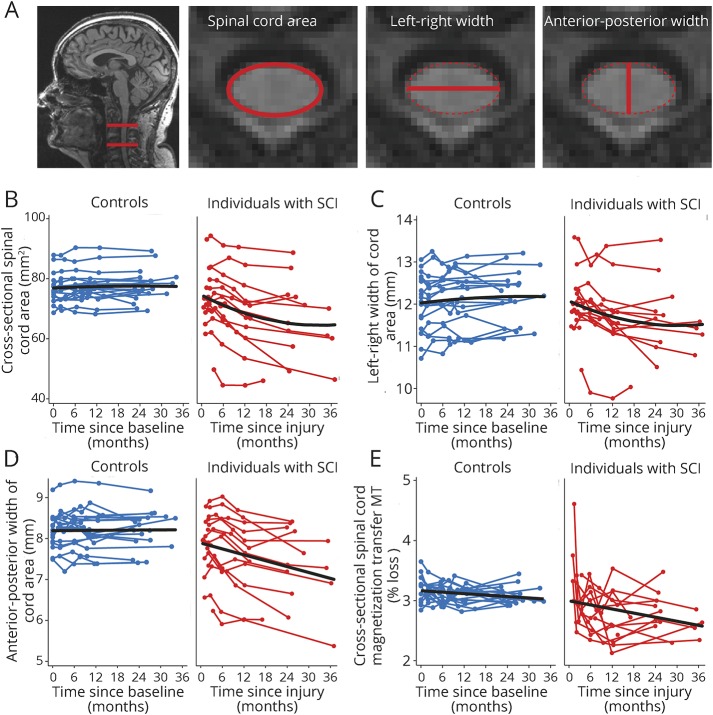
Longitudinal changes in spinal cord MRI indices (A) Illustration of measures of cross-sectional spinal cord area, left-right width, and anterior-posterior width at cervical level C2-C3. (B) Change in cross-sectional spinal cord area at the C2-C3 level and (C) left-right width and (D) anterior-posterior width after injury in patients with spinal cord injury and in controls over 2 years. (E) Change in mean MT (in % loss of magnetization) at the C2-C3 level after injury in patients with spinal cord injury and in controls over 2 years. Note that black, solid lines depict the fitted model; blue and red points and lines show observed individual longitudinal data for controls and patients, respectively. MT = magnetization transfer saturation; SCI = spinal cord injury.

Using an active-surface model (figure 1A), we measured the cross-sectional cord area at the C2-C3 level. This level offers the most reliable assessment site using semi- or fully-automated segmentation methods^16^ (e-Methods, links.lww.com/WNL/A316). This allowed us to extract the anterior-posterior width (APW—elliptical short axis) and the left-right width (LRW—elliptical long axis) of the cord. We also superimposed the region of interest (ROI) corresponding to the cord on MT maps to evaluate the mean MT inside this cervical cord cross-section.

#### Neurodegeneration within the brain over 2 years

Longitudinal image preprocessing for voxel-based morphometry^17,18^ and voxel-based quantification^19^ were used to further assess volumetric and microstructural quantitative brain changes (e-Methods, links.lww.com/WNL/A316).

### Regions of interest

We focused on structural changes within the motor, sensory, and limbic system because these brain areas showed structural changes within the first year following the injury.^5,6^ We defined bilateral ROIs using the anatomy toolbox for statistical parametric mapping (SPM)^20^ to delineate the corticospinal tracts (CSTs) and the union of the primary motor (M1) and sensory (S1) cortices to represent the sensorimotor system. Using the same atlas, we included the bilateral anterior cingulate cortex (ACC), thalamus, secondary somatosensory (S2) cortex, and insula using a single ROI to represent the limbic system. The brainstem and cerebellum were defined as a further ROI using the SUIT toolbox for SPM.^21^

### Statistical analysis

#### Cord

Stata 13 (StataCorp LP, College Station, TX) was used to statistically assess recovery and change in spinal cord MRI indices. Rates of change of cord area, LRW, APW, mean cord MT (all participants), and recovery (patients only) were estimated with linear mixed effects models—with the MRI and clinical measure as response variable. Intercept, study time, log time, and their group interactions were included as predictors to model linear and nonlinear changes specifically for clinical groups. The linear effect corresponds to a progressive change, while a nonlinear effect models a deceleration of markers of neurodegeneration. Finally, regression models identified associations between anatomical changes by 6 months and 2-year clinical outcome measures, adjusting for potentially confounding effects of age and clinical change between 6 months and baseline.

#### Brain

We used SPM12 to analyze group differences of structural trajectories (e-Methods, links.lww.com/WNL/A316, and fil.ion.ucl.ac.uk/spm/ for technical details). We followed a conservative 2-stage summary statistics approach^22^ frequently used in fMRI and longitudinal image analysis.^23^ In the first stage, we used scans at all time points from each participant to estimate individual quadratic trajectory models y(t) = β_0_ + β_1_ t + β_2_ t^2^ and evaluated the intercepts (β_0_), rate of change (β_1_), and quadratic effects (β_2_) for each participant, where t denotes time since injury. In a second stage, we used statistical parametric maps of 2-sample parametric *t* tests (for all voxels within each ROI) to test for group differences while adjusting for age and sex as covariates of no interest. Group differences of linear (e.g., β_1_ < 0 indicating decline) and quadratic (e.g., β_2_ > 0 indicating deceleration) effects were identified as significant using random field theory for correction of multiple comparisons within each considered ROI. Significant clusters were identified after applying a conservative cluster-forming threshold of *p* = 0.001. We additionally used SPM's multiple linear regression models to test for associations among brain changes, lesion level, and clinical recovery in patients adjusting for potentially confounding effects of age, sex, and clinical change between 6 months and baseline. The explanatory variables in these (between-subjects) analyses were lesion level and clinical outcome, while the response variables were changes in structural markers over the first 6 months*.*

## Results

### Patients' characteristics and clinical outcomes

Nine patients were tetraplegic (3 American Spinal Injury Association Impairment Scale [AIS] A “complete”) and 6 paraplegic (3 AIS A “complete”) (table 1). The mean interval to the first scan following injury was 49.67 days (SEM 5.91; range 17–102), to the second scan 103.25 (12.40; 63–193), to the third scan 220.36 (18.69; 158–369), to the fourth scan 389.93 (29.60; 285–723), and to the fifth scan 881.14 (43.07; 718–1,109) days.

**Table 1:**
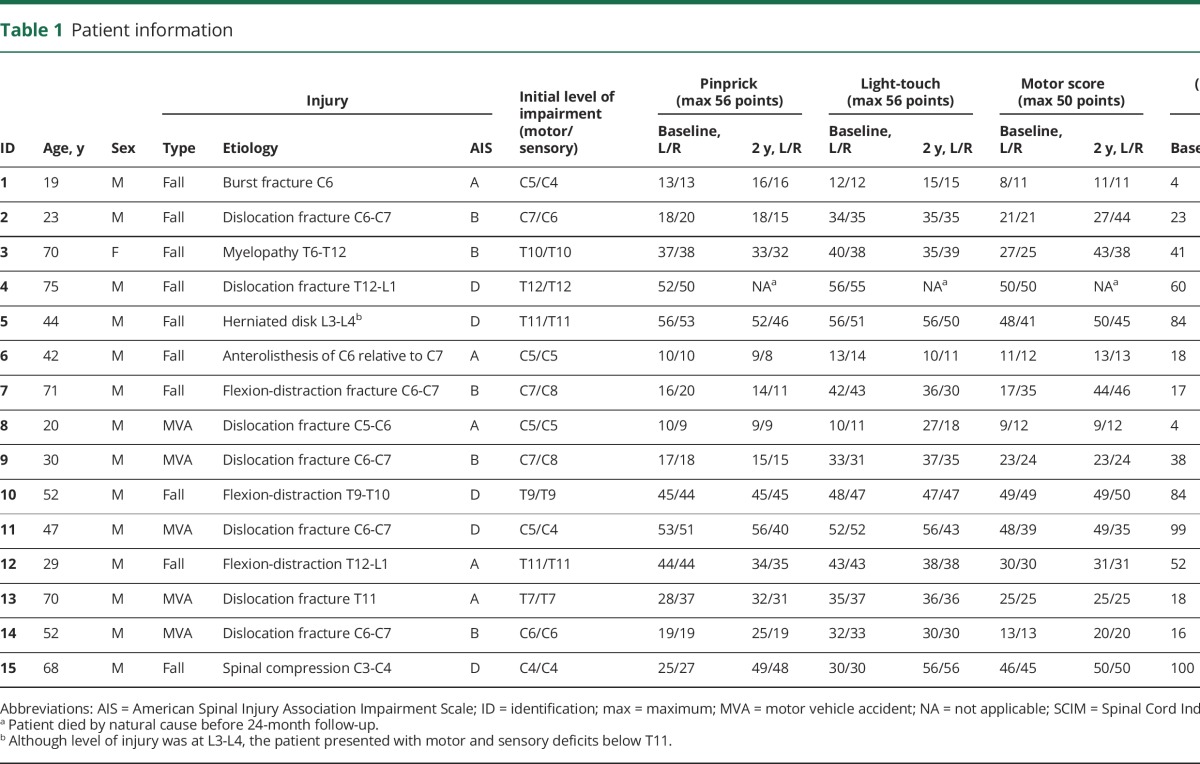
Patient information

Over 2 years, patients recovered by 2.33 points per log month (95% confidence interval [CI] 0.41–4.26) on their lower extremity motor score (*p* = 0.017, all patients), showing weak tendencies to improvement (*p* = 0.135, all patients) by 0.52 points per log month (−0.16 to 1.21) on their upper extremity motor score and by 5.67 points per log month (3.25–8.08) on the SCIM score (*p* < 0.001). Over this time, patients declined by 1.84 points per log month (−3.36 to −0.33) on their pinprick score (*p* = 0.017), and their light-touch score did not show a decline (*p* = 0.851). Neuropathic pain below the level of the lesion emerged in 7 patients, in whom pain intensity increased (*p* = 0.003) by 0.56 points per log month (0.19–0.93).

### Time course of macroscopic cord and brain changes

#### Cord

Over 2 years, a greater linear decline of cord area (*p* < 0.001), APW (*p* < 0.001), and LRW (*p* < 0.001) was found in patients compared to controls (figure 1, B and C). In patients, cord areas decreased by 0.62 mm^2^ per month (*p* < 0.001, 95% CI −0.77 to −0.46), APW decreased by 0.04 mm per month (*p* < 0.001, 95% CI −0.060 to −0.024), and LRW by 0.04 mm per month (*p* < 0.001, 95% CI −0.06 to −0.020) but were unchanged in controls (cord area: *p* = 0.39; APW: *p* = 0.83; LRW: *p* = 0.13). The rate of change of cord area (by 0.01 mm^2^ per month, *p* < 0.001, 95% CI 0.005–0.014) and LRW decrease (by 0.001 mm per month, *p* = 0.011, 95% CI 0.0002–0.001) showed a greater leveling-off (i.e., a positive quadratic effect) over 2 years in patients compared to controls. In controls, we did not detect any nonlinear (quadratic) changes in cord area (*p* = 0.54), APW (*p* = 0.89), and LRW (*p* = 0.42).

#### Brain

**Figure 2:**
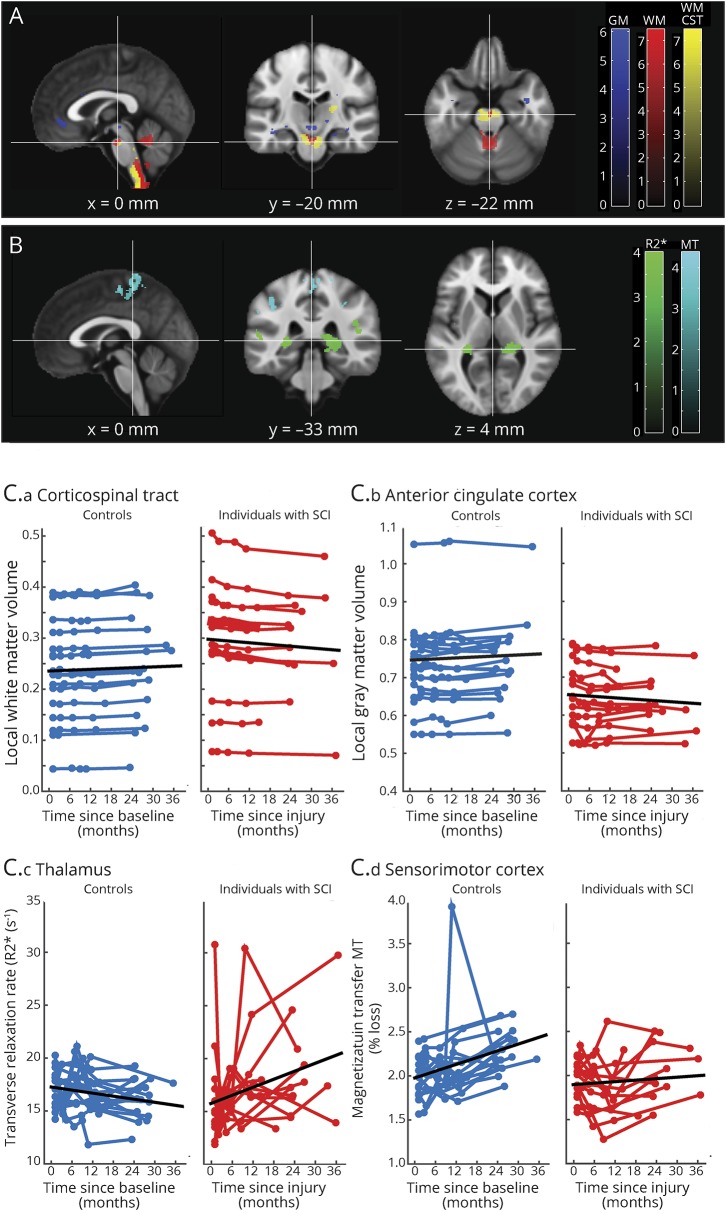
Longitudinal changes in brain volume, myelin, and iron shown by longitudinal voxel-based morphometry (A) and voxel-based quantification (B) (A, B) Overlay of statistical parametric maps (*t* values uncorrected *p* < 0.001, shown for descriptive purposes, masked by the union of regions of interest) showing regions of volume changes in GM volume (in blue), and WM CST volume (yellow), WM excluding the CST (red), MT (cyan), and effective transverse relaxation rate (in green). Corresponding structural trajectories are shown for local effects in voxel-based morphometry and voxel-based quantification ([C.a] CST, [C.b] anterior cingulate cortex, [C.c] thalamus, [C.d] sensorimotor cortex). Note that black, solid lines depict the fitted model; blue and red points and lines show observed individual longitudinal data for controls and patients, respectively. CST = corticospinal tract; GM = gray matter; MT = magnetization transfer saturation; R2* = effective transverse relaxation rate; SCI = spinal cord injury; WM = white matter.

Over 2 years, white matter (WM) volume within the CSTs of patients decreased more rapidly than in controls, with differences in the medulla oblongata, cerebellar peduncle, and right internal capsule. Outside the CSTs, WM volume decreased in the medulla oblongata and cerebellar vermis. Over 2 years, gray matter (GM) volume decreased in the left insula, left ACC, and right thalamus. No GM volume changes occurred in S2 over time. WM volume decreases within the left cerebellum accelerated in patients compared to controls (table 2, figure 2) over 2 years.

**Table 2:**
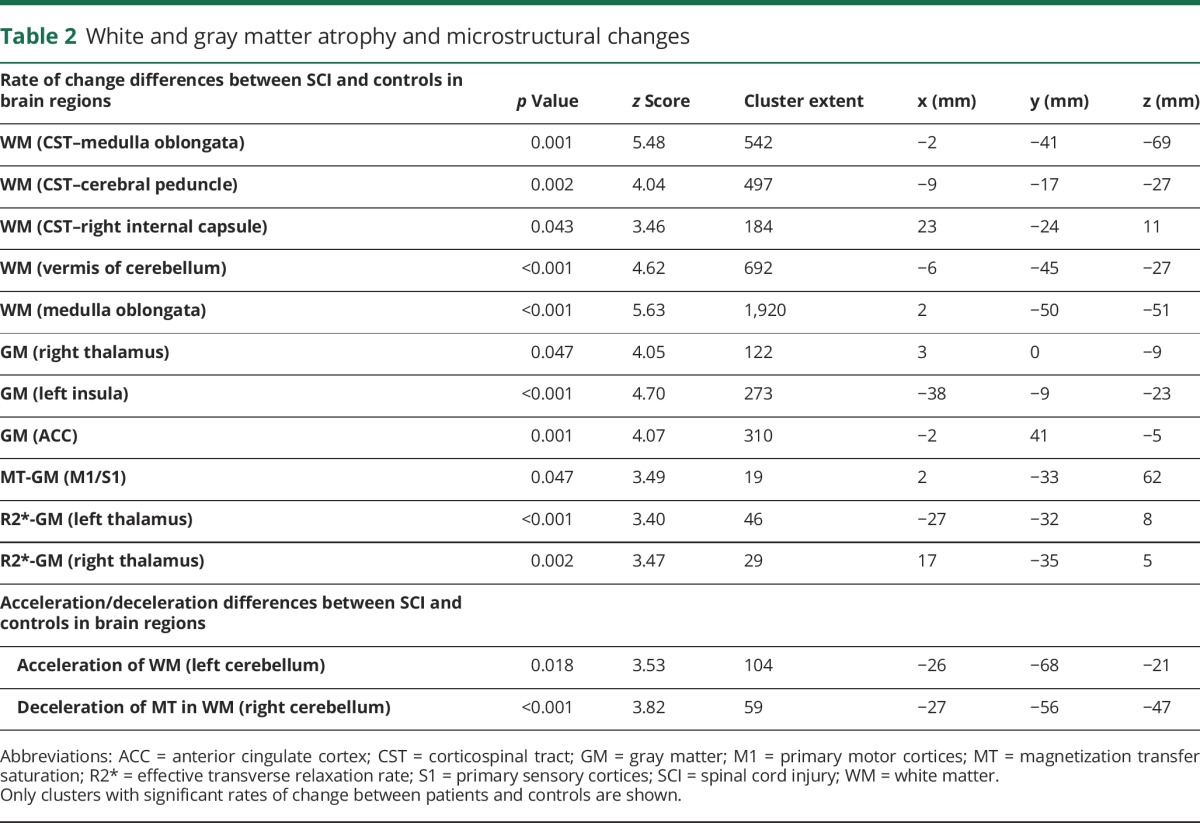
White and gray matter atrophy and microstructural changes

### Time course in microstructural imaging markers

#### Cord

Cord MT decreased in patients relative to controls (*p* = 0.032). In patients, mean cord MT decreased by −0.05% per month (*p* < 0.001, 95% CI −0.07 to −0.02) with no effect seen in controls (*p* = 0.18) (figure 1E).

#### Brain

There were linear MT decreases within the GM of patients relative to controls in the leg area of M1 and linear increases in R2* of GM in the bilateral thalami (figure 2) over 2 years. Testing for partial recovery effects in terms of decelerating changes, we observed a deceleration of the WM MT changes in the right cerebellum over 2 years.

### Effect of rostrocaudal level of spinal injury on neurodegeneration

A higher rostrocaudal level of spinal injury was associated with greater loss in cord area over 2 years (*p* = 0.037, *R*^2^ = 0.42), greater decreases in the MT within GM in the right M1 over 6 months (*p* = 0.006, *z* score = 3.75, cluster extent 27), and greater WM volume loss within the right cerebellum at 6 months (*p* = 0.002, *z* score 3.80, cluster extent 95).

### Prediction of clinical outcomes

**Figure 3:**
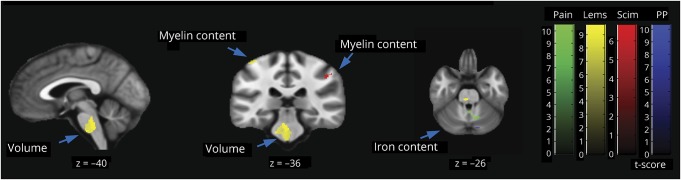
Correlation between brain MRI changes at 6 months and 2-year clinical outcome (*t* values uncorrected *p* < 0.001, shown for descriptive purposes, masked by the union of the regions of interest) Better lower extremity motor score (Lems) (yellow) at 2 years was associated with greater volume (brainstem) and a smaller decrease in MT (sensorimotor cortex) over 6 months. Better functional independence score (Scim) (red) was associated with a smaller decrease in MT in the somatosensory cortex, bilaterally. A worse pinprick (PP) (blue) score at 2 years was associated with increases in R2* in the right cerebellum and right ACC over 6 months. Greater increases in neuropathic pain intensity score (green) was associated with greater R2* increases in the right secondary sensory cortex, in left ACC, and the cerebellum, bilaterally over 6 months. Note that not all clusters are shown, but are presented in Table e-1. ACC = anterior cingulate cortex; MT = magnetization transfer saturation; R2* = effective transverse relaxation rate; SCIM = Spinal Cord Independence Measure.

A better lower extremity motor score at 2 years was associated with a smaller decrease in cord area (figure e-1, links.lww.com/WNL/A314), a smaller decrease in WM volume at the level of the medullary pyramid (i.e., CST) and pons, and a smaller decrease in MT in the sensorimotor cortex, bilaterally over 6 months. A better SCIM score at 2 years was associated with a smaller decrease in MT in the somatosensory cortex, bilaterally over 6 months. A worse 2-year pinprick score was associated with a greater loss in cord area (figure e-1) and an increase in R2* in the right cerebellum and right ACC over 6 months. Greater increases in neuropathic pain intensity were associated with greater R2* increases in the right S2, in left ACC, and the cerebellum, bilaterally over 6 months (table e-2, links.lww.com/WNL/A315; figure 3).

## Discussion

This study characterized progressive changes in macroscopic and microstructural MRI markers of neurodegeneration in the spinal cord, which continue for at least 2 years post trauma. Crucially, while volume changes slow down at the level of injury, both macroscopic and microstructural measures of neurodegeneration show sustained changes, as a linear function of time, in the spinal cord and brain. The magnitude of neurodegeneration at the level of the spinal cord, brainstem, and cortex over the first 6 months predicted clinical outcome at 2 years, independently of early clinical changes.

Signs of neurodegeneration above the level of injury occur with distinct temporal and spatial patterns over the 2 years after acute SCI. At the cord level, the decrease of cord area and LRW, potentially reflecting retrograde changes in the CSTs,^24^ decelerated over 2 years, while the APW, potentially reflecting ongoing wallerian degeneration in the posterior columns,^24,25^ showed sustained changes. Further upstream, atrophy occurred within the motor, sensory, and limbic systems, with acceleration of volume loss within the cerebellum. However, changes in volumetric measures reflect an accumulation of pathologic processes and are therefore insensitive to individual disease processes. We found clear evidence that quantitative markers of myelin and iron change continue over time, reflecting progressive myelin changes^12^ and iron accumulation.^13^ Crucially, sustained changes in myelin-sensitive MT were expressed in the spinal cord and leg area of M1—demonstrating continuous myelin changes within WM and GM^9^ in the absence of macroscopic changes.^26^ This finding is supported by a previous serial diffusion tensor study that showed progressive diffusivity changes across the CST^8^ following acute SCI. Changes in microstructure might also reflect ongoing activity-dependent structural changes (i.e., reorganization) as seen after intensive training.^27^ Of interest, GM atrophy within the thalamus was accompanied by increasing iron levels (i.e., R2*). Such iron accumulation may be indicative of myelin breakdown triggered by oxidative stress and chronic inflammation^28,29^ leading to a release of iron.^30,31^ Within the cerebellum, accelerated volume decline was associated with deceleration in myelin-sensitive MT, reflecting ongoing processes in the context of compensation, decompensation, and the compounding of functional deficits.^2^

The magnitude of neurodegeneration within the spinal cord, cerebellum, and leg area of M1 are driven by the rostrocaudal level of injury because a lesion of the cervical cord affects the structural integrity of a higher number of fibers and neurons than a comparable thoracic lesion.^24,7^ Larger cohorts and longer time intervals might offer further clues as to whether the rate of neurodegeneration can be predicted by the level and severity of injury. Finally, inflammation^29^ leading to subsequent swelling of neuronal tissue might have biased morphometric measurements toward initial increases in area and volume. To resolve this issue, future serial investigation needs to sample at higher frequency to disentangle the relationship between inflammation (i.e., tissue swelling) and atrophy (i.e., tissue shrinkage), informed by monitoring inflammatory markers in the CSF.^32^

Clinical recovery occurs most rapidly within the first 6 months and levels off, at the latest, after 2 years.^1^ We used changes in the macroscopic neuronal tissue compartments, together with measures of myelination and iron content, to provide greater insight into the complicated relationship between neuronal changes^33,34^ and clinical recovery. While clinical recovery leveled off at 2 years, neurodegeneration continues beyond this point. Of note, we show a close association between slower longitudinal changes in the progression of macroscopic and microstructural integrity and better 2-year clinical outcome, independent of early clinical changes. Less neuropathic pain was related to less myelin reduction and less iron accumulation in the brain areas that have been previously identified as hotspots for altered pain processing in patients with chronic SCI.^7^ The fact that these associations hold their significance after correcting for the initial clinical changes suggests that the neuroimaging biomarkers significantly adds to the predictive potential of clinical measures of recovery.^35^ However, these early MRI changes cannot inform treatment regimens that could improve patient care during their hospital stay.

This study has some limitations. It is worth mentioning that this explorative study is based on observing patients without a priori power calculation. However, we hope that our findings on SCI disease progression might inform more comprehensive prospective studies in near future. Despite existing histologic evidence that both MT and R2* markers associate with their biochemical counterparts,^12,13^ they remain indirect markers of myelin and iron deposition. We therefore cannot exclude a partial contribution of unexplored physiologic/cellular processes occurring after SCI. Moreover, unobserved latent lifestyle or genetic factors differentiating patients with SCI from controls a priori cannot be precluded based on applied standardized neurologic tests. Because of the serious clinical condition of the neurologic patients, the image acquisition was delayed by about 50 days on average. This clearly limited our ability to explore early injury-induced neuronal changes. However, our goal to assess mid- to long-term progression trends over years is unlikely to be affected by this short-term shift (of typically less than 6%) of the overall longitudinal acquisition interval. Our sample size was small, but compliance was very good given the severity of the disorder. Effectively, this resulted in 4.7 MRI scans per person, which is comparably high for longitudinal neuroimaging studies. The potentially confounding effect of upgrading the MRI scanner is mitigated by virtue of acquiring data from patients and controls before and after upgrade, allowing us to account for the upgrade effect (common to both cohorts). Given the fact that we were using novel imaging markers, we were not able to assess the extent of measurement error for the outcome measures.^36–39^ However, this does not invalidate our presented group comparisons and significant findings: it is possible that, in the future, variability caused by measurement error may be reduced, in which case requisite sample sizes would be smaller, reflecting the reduced measurement noise. Crucially, our results were based on the group level analysis. Given that image-based markers are noisy, considering, for example, a voxel or ROI of one image modality separately, limits the potential for accurate predictions about individuals. In the future, we therefore hope to exploit the potential of multivariate methods for accurate individual-level clinical predictions of disease progression using multimodal and spatially distributed patterns in larger, pooled SCI samples.^40^

Our findings illustrate the progressive and enduring neurodegenerative and plastic processes induced by SCI, highlighting a temporally structured neurodegenerative process that encompasses the spinal cord and brain.^2^ The changes revealed by neuroimaging are sufficiently large, systematic, and predictive to render them viable candidates for assessing the effects of treatment, including rehabilitation.

## Glossary

ACC: anterior cingulate cortex
AIS: American Spinal Injury Association Impairment Scale
APW: anterior-posterior width
CI: confidence interval
CST: corticospinal tract
GM: gray matter
LRW: cord left-right width
M1: primary motor cortices
MT: magnetization transfer saturation
R2*: effective transverse relaxation rate
ROI: region of interest
S2: secondary sensory cortices
SCI: spinal cord injury
SCIM: Spinal Cord Independence Measure
SPM: statistical parametric mapping
WM: white matter
